# Supplementary material for: Mussel biofiltration effects on attached bacteria and unicellular eukaryotes in fish-rearing seawater
Source: PeerJ. 2016 Mar 29;4:e1829. doi: 10.7717/peerj.1829 (PMC4824906; doi:10.7717/peerj.1829)
Supplement: Supplemental Information 1 [file peerj-04-1829-s001.docx]

**Mussel biofiltration effects on attached bacteria and unicellular eukaryotes in fish rearing seawater**

E. Voudanta, K. A. Kormas, S. Monchy, A. Delegrange, D. Vincent, S. Genitsaris, U. Christaki

Supplementary material

**Table S1**. Description of environmental parameters. Mean (± SD) and range are presented for all of the sampling dates for Control (C) and Test (T) tanks (data from Delegrange et al. 2015).

|  | **T**  **(°C)** | **Salinity**  **(PSU)** | **Dissolved O_2_**  **(mg L^-1^)** | **Turbidity (NTU)** | **Chl-a**  **(µg L^-1^)** |
| --- | --- | --- | --- | --- | --- |
| **CONTROL** | 20.98±1.94  (18.12-23.7) | 33.85±0.43  (32.94-34.26) | 8.15±4.14  (4.7-20) | 4.09±1.04  (2.58-5.97) | 4.97±3.80  (0.80-10.36) |
| **TEST** | 20.82±1.71  (18.22-22.94) | 33.58±0.40  (32.81-33.97) | 9.71±4.85  (3.4-18.3) | 1.37±0.73  (0.64-2.91) | 0.79±0.62  (0.16-2.28) |


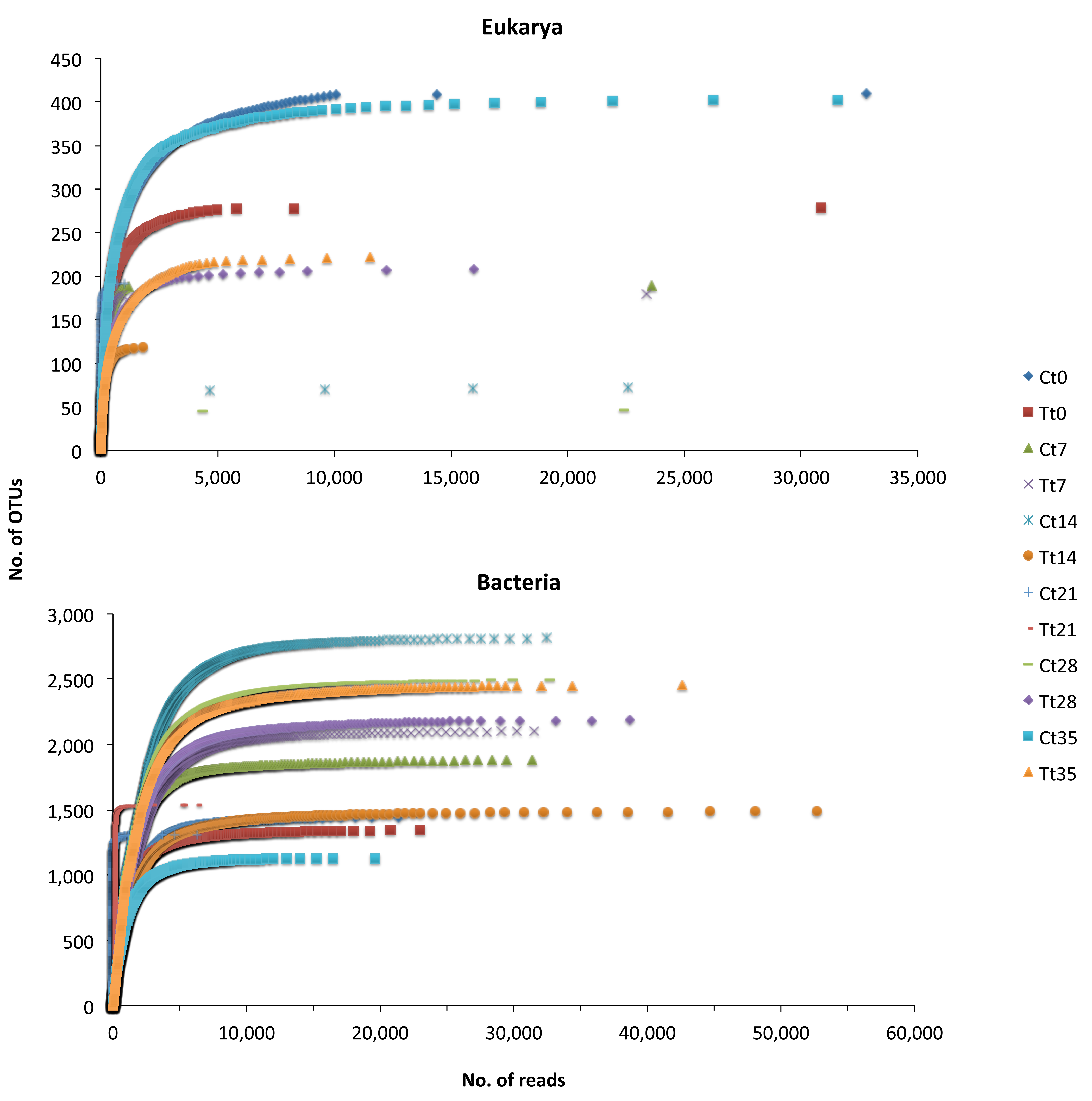


**Figure S1**. Rarefaction curves of the eukaryotic and bacterial operational taxonomic units (OTUs) in the control (C) and mussels tanks (T) at all sampling dates.


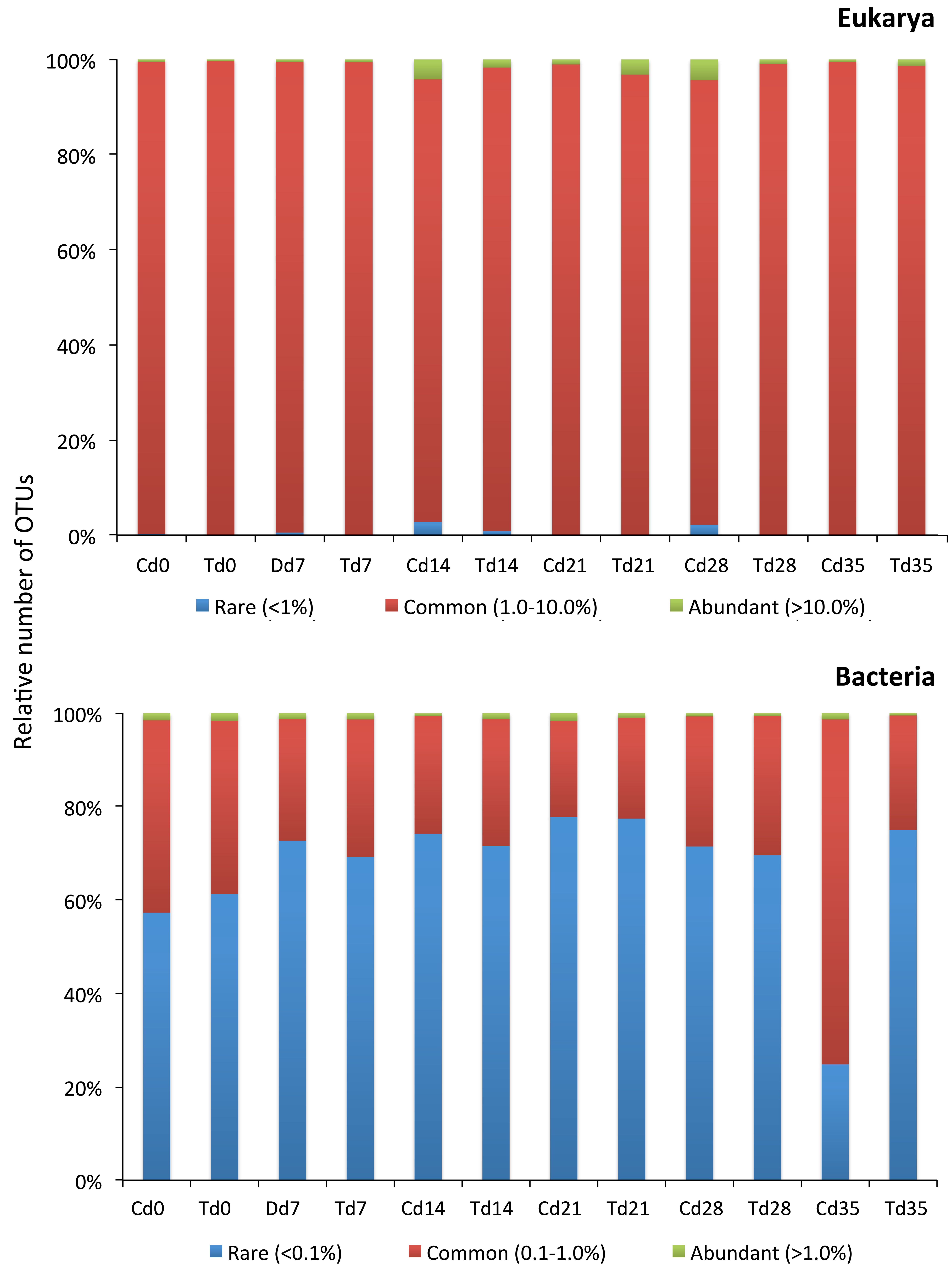


**Figure S2**. Changes in the relative abundance of rare, common and abundant operational taxonomic units (OTUs) in the control (C) and mussels tanks (T) at all sampling dates.


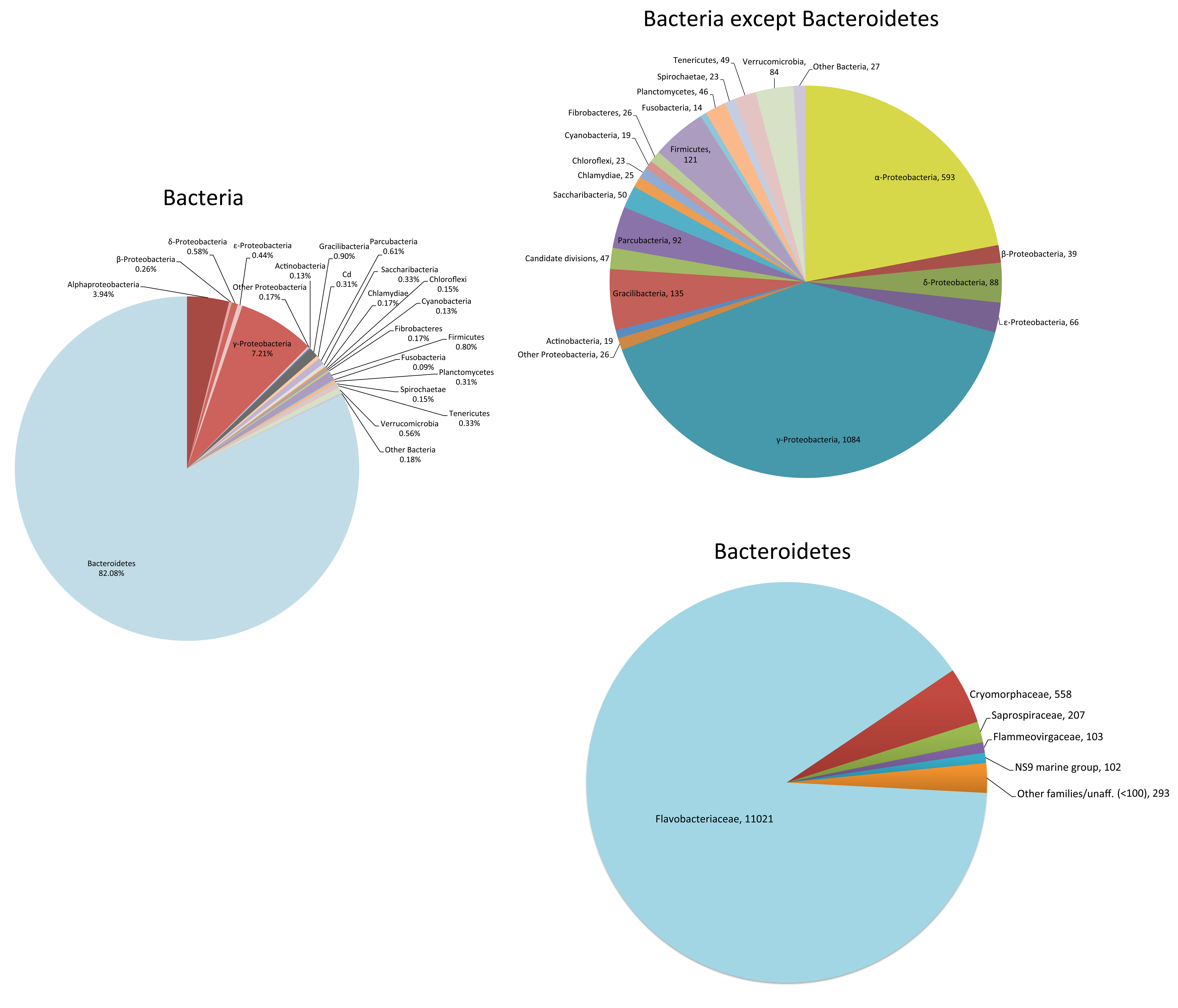


**Figure S3**. Relative abundance of the bacterial operational taxonomic units in the control (C) and mussels tanks (T) at all sampling dates.


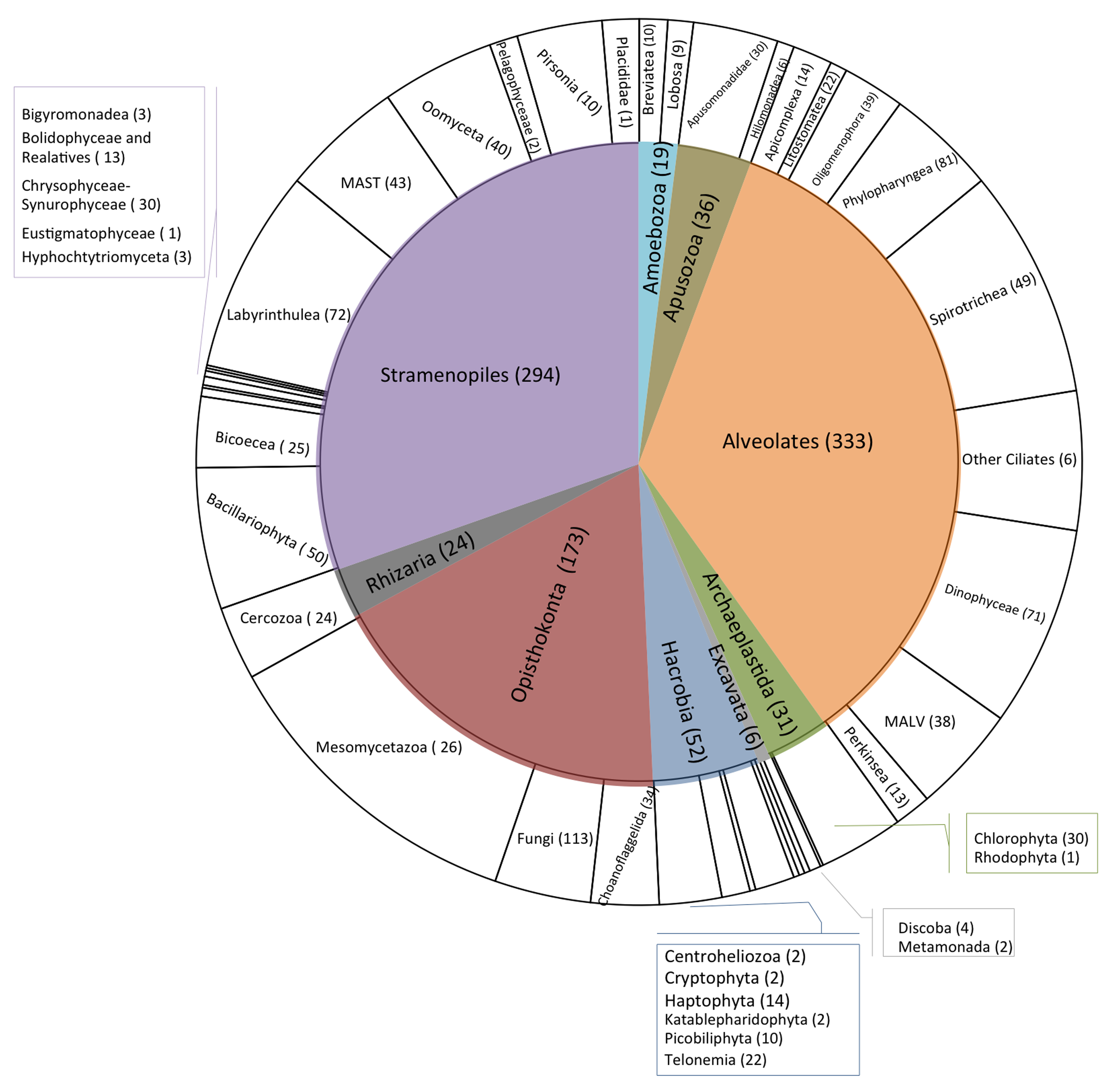


**Figure S4**. Relative abundance distribution of the unicellular eukaryotic operational taxonomic units (OTUs) in the control (C) and mussels tanks (T) at all sampling dates.
